# Phonocardiogram Signal Processing for Automatic Diagnosis of Congenital Heart Disorders through Fusion of Temporal and Cepstral Features

**DOI:** 10.3390/s20133790

**Published:** 2020-07-06

**Authors:** Sumair Aziz, Muhammad Umar Khan, Majed Alhaisoni, Tallha Akram, Muhammad Altaf

**Affiliations:** 1 Department of Electronics Engineering, University of Engineering and Technology Taxila, Taxila 47080, Pakistan; sumair.aziz@uettaxila.edu.pk; 2 College of Computer Science and Engineering, University of Ha’il, Ha’il 55211, Saudi Arabia; majed.alhaisoni@gmail.com; 3 Department of Electrical and Computer Engineering, COMSATS University Islamabad, Wah Campus, Wah Cantonment 47040, Pakistan; mohammadaltaf@gmail.com

**Keywords:** phonocardiogram, machine learning, empirical mode decomposition, feature extraction, mel-frequency cepstral coefficients, support vector machines, computer aided diagnosis, congenital heart disease, statistical analysis

## Abstract

Congenital heart disease (CHD) is a heart disorder associated with the devastating indications that result in increased mortality, increased morbidity, increased healthcare expenditure, and decreased quality of life. Ventricular Septal Defects (VSDs) and Arterial Septal Defects (ASDs) are the most common types of CHD. CHDs can be controlled before reaching a serious phase with an early diagnosis. The phonocardiogram (PCG) or heart sound auscultation is a simple and non-invasive technique that may reveal obvious variations of different CHDs. Diagnosis based on heart sounds is difficult and requires a high level of medical training and skills due to human hearing limitations and the non-stationary nature of PCGs. An automated computer-aided system may boost the diagnostic objectivity and consistency of PCG signals in the detection of CHDs. The objective of this research was to assess the effects of various pattern recognition modalities for the design of an automated system that effectively differentiates normal, ASD, and VSD categories using short term PCG time series. The proposed model in this study adopts three-stage processing: pre-processing, feature extraction, and classification. Empirical mode decomposition (EMD) was used to denoise the raw PCG signals acquired from subjects. One-dimensional local ternary patterns (1D-LTPs) and Mel-frequency cepstral coefficients (MFCCs) were extracted from the denoised PCG signal for precise representation of data from different classes. In the final stage, the fused feature vector of 1D-LTPs and MFCCs was fed to the support vector machine (SVM) classifier using 10-fold cross-validation. The PCG signals were acquired from the subjects admitted to local hospitals and classified by applying various experiments. The proposed methodology achieves a mean accuracy of 95.24% in classifying ASD, VSD, and normal subjects. The proposed model can be put into practice and serve as a second opinion for cardiologists by providing more objective and faster interpretations of PCG signals.

## 1. Introduction

Congenital heart disease (CHD) is one the most common birth defects which affect the overall structure of the heart and vessels, found in not more than 1% of newborns [1]. CHD manifests itself at birth and symptoms may vary from mild asymptomatic cases to severe, life-threatening indications. With advances in treatment, there is an increasing population of adults surviving with congenital heart malformations. Globally, cardiovascular diseases (CVD) are the main cause of mortality. Many adult CHD survivors presenting an increased risk of CVD [2] may have long term health problems, which affect their quality of life. In Pakistan, CHD remains to be an important medical issue and the number of patients is increasing every day [3]. Among newborn children and youngsters, cardiac disorders are responsible for a large extent (30% to 50%) of mortality brought about by birth surrenders. The most common cardiac defects which represent about 85% of all congenital heart diseases are ventricular septal defects (VSDs; 34%), and atrial septal defects (ASDs), which contribute up to 13% [4]. Like any other medical issue, robust diagnosis methods are required for the timely diagnosis of the CHDs. Different non-obtrusive procedures are utilized in identifying heart defects. Using the electrocardiogram (ECG) is one of the most common paths for identifying heart issues; it is based on the electrical signals generated during the heart muscle contraction/relaxation. The ECG reveals the electrical activity of the heart and is mostly recorded by the placement of three electrodes for early diagnosis. It comprises five waves; i.e., P, Q, R, S, and T. These waves are prepared to make sense of different pathologies [5].

Another commonly used mechanism for diagnosis of heart disorder is through the analysis of the heart sound [6]. Easy access to digital stethoscopes allows medical staff to record and analyze heart sounds for diagnostic purposes. The phonocardiogram (PCG) records heart sounds and murmurs in the form of a plot and the machine by which these sounds are recorded is known as the phonocardiograph. It is one of the non-obtrusive systems, which records heart condition in audible form. Heart sounds are generated by the opening or closing of the heart valves. Blood flow through the valves’ orifices or into the ventricular chambers also produces heart sounds. Recording of the PCG signal consists of four important heart sound constituents; namely, S1, S2, S3, and S4.

An atrial septal defect (ASD) [7] is a birth deformity of the heart in which there is a hole in the wall (septum) that isolates the upper chambers (atria) of the heart. A gap can fluctuate in size and requires a medical procedure. The reasons for CHD amongst most infants are obscure, but genetic factors are also important, as a few infants have heart defects as a result of changes in their genes or chromosomes [8]. A ventricular septal defect (VSD) is an opening in the heart, a typical heart imperfection that is present during childbirth (congenital).

Extensive research has been carried out for the detection and classification of congenital heart disorders using the PCG signal. The PCG signal classification approach was suggested using the nested set of classifiers; namely, random forest, cost-sensitive classifier, and LogitBoost (LB) [9]. A combination of time domain, statistical, and frequency domain features was used for effective classification. Cepstrum-analysis-based feature extraction was performed to classify normal and abnormal PCG signals through a support vector machine (SVM) classifier [10].

PCG signal classification was achieved through linear SVM and a combination of dynamic time wrapping (DTW) and Mel-frequency cepstral coefficient (MFCC) features in [11] to achieve 82.4% accuracy. The screening method of PCG signals using a modified Arash-band method and an SVM classifier has been used [12]. In [13], the PCG signal was first segmented into S1, systole, S2, and diastole through the hidden Markov model (HMM). Gammatone frequency cepstral coefficient (GFCC) features were extracted to perform classification using weighted SVM without segmentation and with segmented signals. The sensitivity of 90.3% and specificity of 89% were achieved through 10-fold cross-validation. Rubin et al. [14] proposed a method for classification of normal and abnormal PCG signals based on Mel-frequency cepstral coefficients (MFCCs) and a two-layer convolutional neural network (CNN). This method achieved an overall score of 83.99% with the PHY16 challenge database. Spectrogram features from PCG were used to train CNN and Adaboost classifiers [15]. A simple decision rule was implemented on outputs of both classifiers to generate final classification results with an overall reported accuracy of 89%. In another study [16], the authors used a Hamming filter for noise reduction in PCG signals. A four-layer 1D CNN for PCG signal classification was employed and the overall accuracy of this method was 79%. In a recent study [17], the CNN architecture was presented for heart sound classification. CNN was tested on different feature sets, such as Mel-Spectrogram, MFCC, and sub-band envelopes.

Zhihai Tu et al. performed filtration of heart sound signals using wavelet transform. Heart sound segmentation was performed using Hilbert transform [18,19], and cubic polynomial interpolation [20]. Samuel E Schmidt et al. presented an easy and cheap system for the identification of coronary artery disease (CAD) using acoustic features. A quadratic discriminant function was used to combine the different features. The accuracy to diagnose the CAD disease is 73% [21]. In another study [22], tunable Q-wavelet transformation [23,24,25] and signal second difference with the median filter were used for the detection of artifact in heart sound. In [26], the classification of heart sound was achieved through power MFCC features fused with fractal features. The nearest neighbor classifier was employed to perform classification. The overall accuracies achieved on three publicly available datasets were 92%, 81%, and 98%. In [27] heart sounds classification was performed through MFCC and linear predictive coding (LPC) features in conjunction with the Adaboost ensemble classifier. In [28], the authors used the least square support vector machine (LSSVM) with wavelet features for the detection of heart pathologies. VSD was diagnosed from the time-frequency feature matrix acquired from heart sounds [29]. The ellipse-based model achieved max accuracy of 97.6% on large VSD sounds. The authors used the auscultation jacket to detect heart abnormalities [30]. The system with a feed-forward neural network as the classifier achieved sensitivity and specificity of 84% and 86% respectively. In [31], normal and abnormal cardiac sounds were classified using ensemble EMD, auto-regressive models, and a neural network. The method showed sensitivity and specificity of 82% and 88% respectively. An efficient method for the detection of abnormal PCG signals was proposed [32] using MFCCs and SVM with a classification accuracy of 92.6%. Classification of CAD and non-CAD subjects from PCG and ECG [33] using a dual input neural network (DINN) achieved specificity, accuracy, and G-mean of 89.17%, 95.62%, and 93.69%, respectively. A combination of machine learning and a deep learning model [34] for identification of congestive heart failure (CHF) from audio PCG obtained an accuracy of 93.2%.

Classification of ASD and normal PCG signals collected from newborn subjects was performed using a combination of short-time Fourier transform (STFT) and MFCC and its derivatives features [35]. Accuracy of 93.2% was achieved through the KNN classifier. An approach based on discrete wavelet transform (DWT) and multilayer perceptron (MLP) for estimation of VSD were presented in [36]. Features such as power, standard deviation, skewness, kurtosis, and Shannon entropy were extracted from eight levels of detailed coefficients of DWT. In another similar study [37], a combination of wavelet and MFCC features was proposed to achieve 97% accuracy on normal and four abnormal classes of heart sounds. In [38], a comparative analysis of four features reduction methods for PCG signals is presented. Experiments were performed on normal patients, and those with three different classes of heart disorders; namely, ASD, VSD and AS. Double discriminant embedding (DDE), feature space discriminant analysis (FSDA), clustering-based feature extraction (CBEF), and feature extracting using attraction points (FEUAP) were used with a KNN classifier. Table 1 presents a comparative summary of existing literature in terms of feature extraction and classification methods and the number of classes used in the experimentation.

In the present research, a novel method for PCG signal analysis for the detection and classification of congenital heart diseases is presented. Classification of ASD and VSD based on PCG signals is targeted using empirical mode decomposition (EMD) and a fusion of MFCC and temporal features. Specifically, a new feature fusion-based approach for the classification of ASD and VSD using PCG signal analysis is proposed. The classification performances of MFCCs and temporal features 1D local texture patterns (1D-LTPs) were individually evaluated and followed by the evaluation over the proposed fused feature representation. The proposed method was shown to be accurate, reliable, and robust due to comprehensive PCG signal representation with reduced features.

The rest of this article is organized as follows. Section 2 describes details about the data acquisition and the proposed methodology. Section 3 presents results of detection and multiclass experiments. A comparative analysis of this work with previous studies is presented in Section 4. In Section 5, conclusions of this research and future directions are described.

## 2. Materials and Methods

### 2.1. Overview

**Figure 1:**
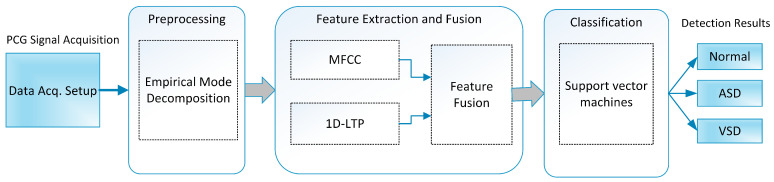
Sketch of the proposed cardiac disorder classification system.

A PCG signal acquired using a stethoscope was digitized through an analog-to-digital converter. Signal preprocessing was performed on the acquired signal to remove possible noise and distortions. A data-driven approach known as empirical mode decomposition (EMD) was applied to denoise the signal. After preprocessing, feature extraction was performed to capture the most significant and decisive information from different classes of PCG signals. MFCC and temporal features were extracted and fused to better represent the signal. Finally, the support vector machine classifier was employed to distinguish different classes of PCG data. A sketch of the proposed system is presented in Figure 1.

### 2.2. Materials

One of the main challenges in studies related to the CHDs is the availability of respective PCG signals. There are several PCG signal datasets available [40,41], but they have following shortcomings.

1. The number of observations (signals) is limited.
2. Not recorded in a hospital environment.
3. Limited to two classes of data; namely, normal and abnormal.

Therefore, a new dataset of PCG signals was acquired that contains ASD, VSD, and normal data classes.

A self-built and low-cost data acquisition system (a microphone fitted in simple stethoscope) was utilized and connected with a computer for the acquisition of PCG signals in.wav format with 16-bit resolution and a sampling frequency of 44.1 kHz. PCG signal data were acquired by placing a stethoscope between the third and fourth left intercostal space. This site is best known for the detection of CHDs through auscultation.

**Figure 2:**
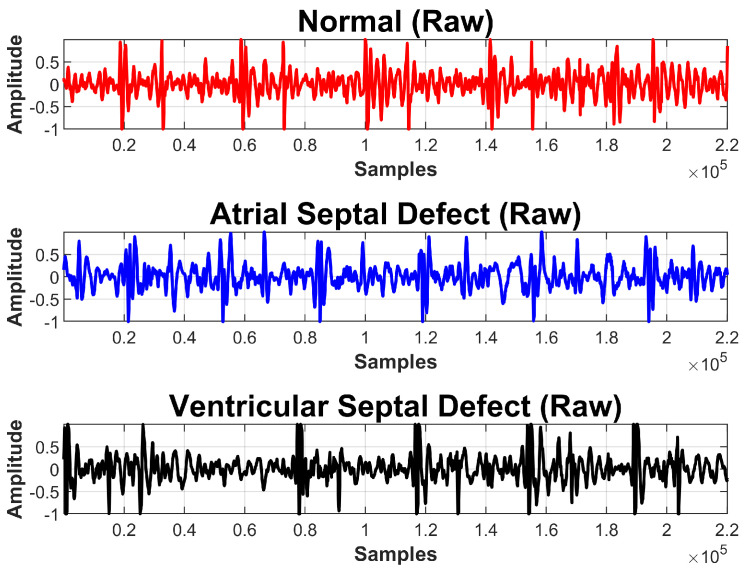
PCG signals collected from normal, arterial septal defect (ASD), and ventricular septal defect (VSD) subjects.

PCG data were acquired from different patients admitted at Rawalpindi Institute of Cardiology, Rawalpindi, Pakistan; 85, 55, and 140 samples were collected from ASD, VSD, and normal subjects respectively. All recordings, each of five seconds, were taken in the hospital environment and under the supervision of an expert physician from the pulmonic, aortic, mitral, and tricuspid areas of the human heart. Labeling of the samples was done by an expert cardiologist who further validated through various tests of each participating subject. Table 2 provides a summary of the dataset according to each class, and examples of signals collected from normal, ASD, and VSD subjects are shown in Figure 2.

The reader may also be interested in the MATLAB codes of the newly developed feature extraction process [42]. However, it only provides experimental results on the PCG dataset comprised of the normal, ASD, and VSD classes.

### 2.3. Preprocessing-Empirical Mode Decomposition

Acquired PCG signal gets corrupted due to embedded electronics, environmental noise, and other body organ artifacts. These noise elements suppress useful discriminative data associated with different classes of cardiac health and thus make the classification process more challenging. Signal denoising is a crucial preprocessing phase to obtain the unique region of interest for each data class, i.e., ASD, VSD, and normal. Empirical mode decomposition (EMD) [43,44,45] is a widely employed method in the domain of medical signal processing for denoising [46,47] and feature extraction [48,49]. EMD reduces the given data into a collection of subcomponents called intrinsic mode functions (IMFs). The process of IMF extraction is known as sifting. The original signal q(t) can be expressed in terms of IMFs and residual signal r(t) as follows:

(1) $$q\left(t\right)=\sum_{k=1}^{N}h_{k}\left(t\right)+r\left(t\right)$$

where the number of extracted IMFs is represented by N and IMFs $h_{k}\left(t\right)$ are obtained from raw PCG signal q(t) through an iterative process known as sifting. Major computing steps of the sifting process are listed below [50].

1. Calculate local minima and maxima from PCG signal q(t).
2. Cubic spline interpolation is performed on local minima and maxima to form lower envelope $e_{min}\left(t\right)$ and upper envelope $e_{max}\left(t\right)$.
3. Calculate the mean of upper and lower envelopes as described by Equation (2).
  (2) $$a\left(t\right)=\frac{1}{2}\left(e_{\min}\left(t\right)+e_{\max}\left(t\right)\right)$$
4. Subtract a(t) from the original signal q(t) as:
  (3) y(t)=q(t)−a(t)
5. Repeat the steps (1)–(4) until the above mentioned two conditions of IMF are fulfilled.

Here, first, IMF is represented as $h_{1}\left(t\right)=y\left(t\right)$. Remaining IMFs from the residual signal are extracted as defined by Equation (4).

(4) $$r_{1}\left(t\right)=q\left(t\right)-h_{1}\left(t\right)$$

**Figure 3:**
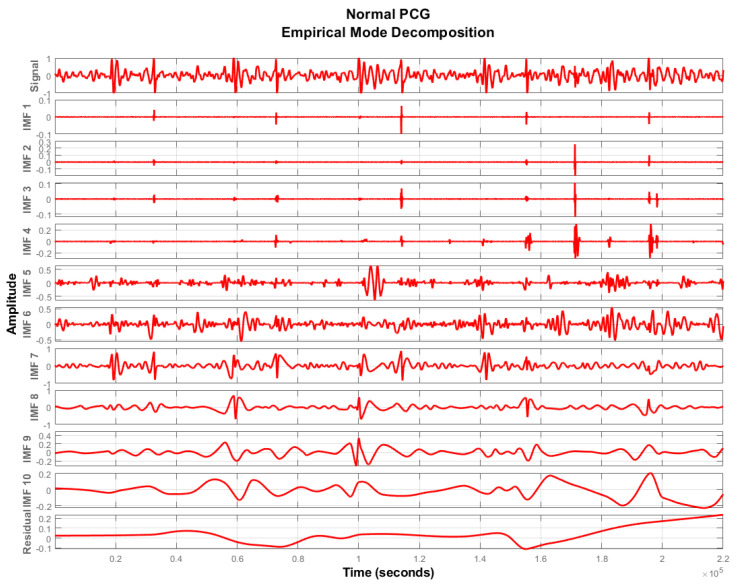
Intrinsic mode functions (IMFs) extracted from the PCG signal of a normal subject.

**Figure 4:**
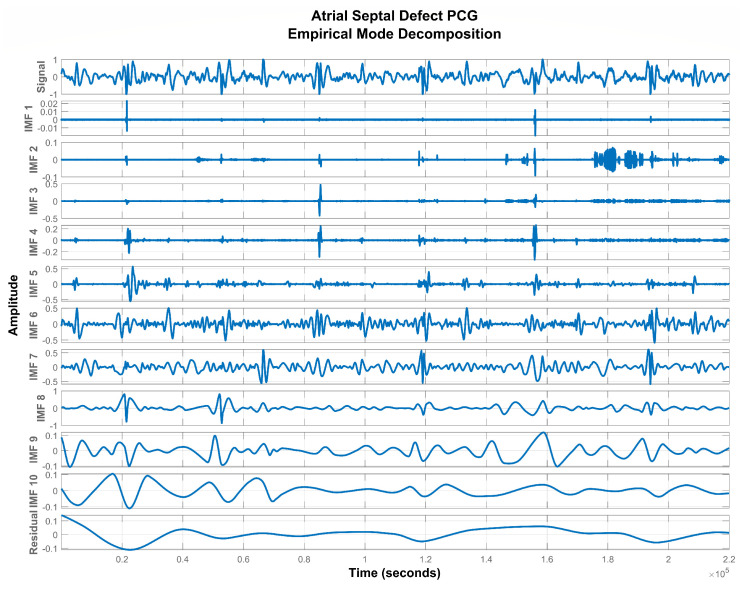
IMFs extracted from the PCG signal of an ASD subject.

**Figure 5:**
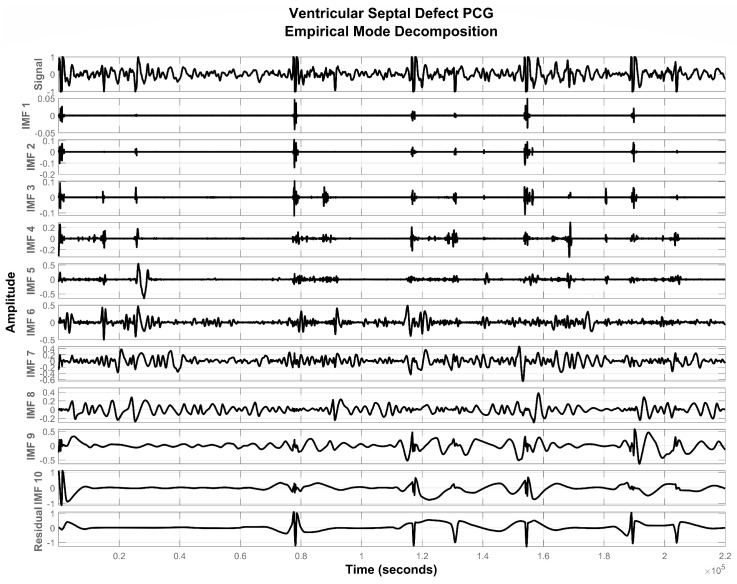
IMFs extracted from the PCG signal of a VSD subject.

To extract the remaining IMFs, $r_{1}\left(t\right)$ is now treated as a new signal and the sifting procedure is iteratively applied until a residual signal becomes monotonic functions. Figure 3, Figure 4 and Figure 5 show IMFs extracted from PCG signals of normal, ASD, and VSD subjects. It was experimentally observed that the first and last two IMFs contain high-frequency noise and DC offset respectively. Therefore, they were subtracted from the remaining signal to acquire a good quality denoised signal represented by x(t) as follows:

(5) $$x\left(t\right)=\sum_{k=2}^{N-2}h_{k}\left(t\right)$$

**Figure 6:**
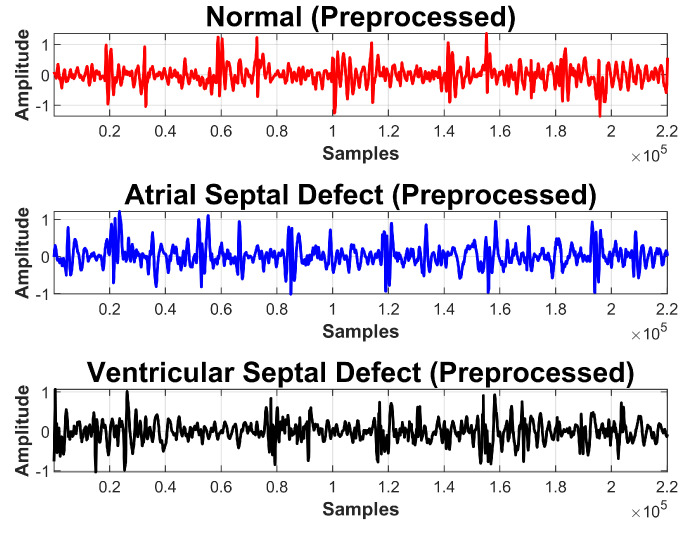
Preprocessed PCG signal of normal, ASD, and VSD subjects.

Figure 6 illustrates the preprocessed signal x(t) for normal, ASD, and VSD subjects.

### 2.4. Feature Extraction

In this step, feature extraction was performed on the preprocessed PCG signal x(t). Frequency-based features such as Mel-frequency cepstral coefficients (MFCCs) and temporal features 1D local texture patterns (1D-LTPs) were extracted. The final feature vector was constructed by fusion of these two feature sets to best represent the PCG signal data of different classes with minimum possible values.

#### 2.4.1. 1D Local Ternary Patterns (1D-LTPs)

**Figure 7:**
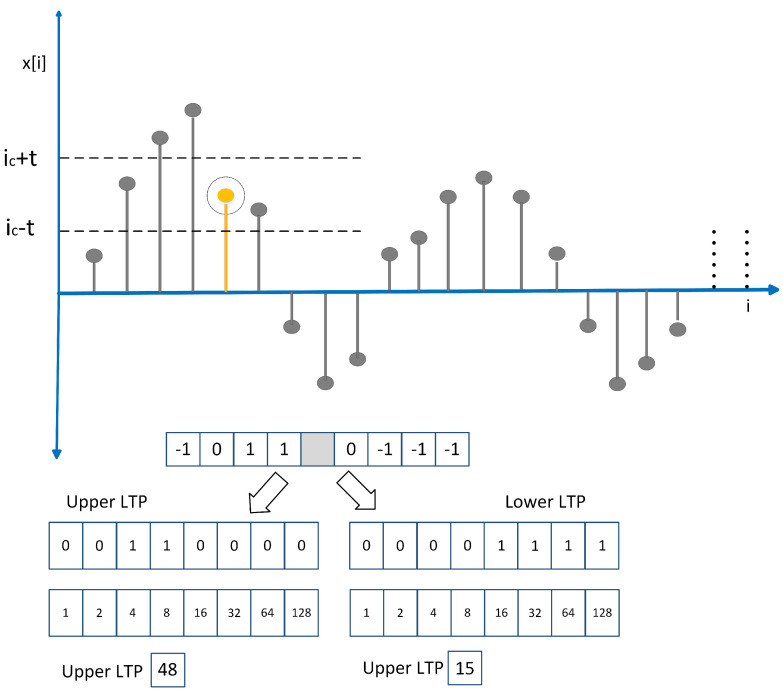
One-dimensional local ternary pattern (1D-LTP) feature extraction steps.

Local ternary patterns are an extended form of widely used temporal features known as local binary patterns [51] used extensively in the domain of computer vision [52,53,54]. One-dimensional local ternary patterns (1D-LTPs) are modified feature descriptors applied for signal processing applications [55,56,57,58]. Steps for extraction of 1D-LTP features are delineated in Figure 7.

To extract 1D-LTP features from preprocessed signal x(t), it is first divided into windows of size W+1. The center sample of each window is θ, the upper bound is θ+ϕ and the lower bound is θ−ϕ. Each window of size W+1 is divided into left and right equal-sized frames around center sample x[i].

(6) $$F\left(x_{i},\theta ,\phi \right))=\left\{\begin{array}{cc} +1, & x_{i}-\left(\theta +\phi \right)\geq 0\\ 0, & \left(\theta +\phi \right)<x_{i}<\left(\theta -\phi \right)\\ -1 & x_{i}-\left(\theta -\phi \right)\leq 0 \end{array}\right.$$

The F(.) is the three-valued vector output having values +1, 0 and −1. F(.) is split into upper and lower patterns using Equations (7) and (9).

(7) $$LTP_{upper}=\sum_{p=1}^{8}S_{u}\left(F(p)\right).2^{p}$$

(8) $$S_{u}=\left\{\begin{array}{cc} 1, & \mathrm{if}\;F(p)=1\\ 0, & \mathrm{otherwise}. \end{array}\right.$$

(9) $$LTP_{lower}=\sum_{p=1}^{8}S_{l}\left(F(p)\right).2^{p}$$

(10) $$S_{l}=\left\{\begin{array}{cc} 1, & \mathrm{if}\;F(p)=-1\\ 0, & \mathrm{otherwise}. \end{array}\right.$$

$LTP_{upper}$ is calculated by using Equation (8) and $LTP_{lower}$ is computed from Equation (10). $LTP_{upper}$ and $LTP_{lower}$ were the resultant LTP feature vectors extracted from the PCG signal.

#### 2.4.2. Mel Frequency Cepstral Coefficients (MFCC)

Mel-frequency cepstral coefficients (MFCCs), a well-known group of features for speech/speaker recognition systems, have recently gained importance as features for classifying heart sounds [26,32,59,60]. Mel frequencies are grounded in the nonlinear physiognomies of the human ear’s sensitivity to different frequencies [61]. MEL frequency is related to linear frequency in Equation (11).

(11) $$Mel\left(f\right)=2595\log_{10}\left(1+\frac{f}{700}\right)$$

**Figure 8:**
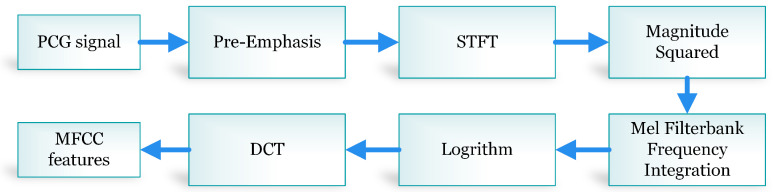
The process of mel-frequency cepstral coefficient (MFCC) feature extraction.

The process of MFCCs’ calculation is shown in Figure 8. The preprocessed PCG signal is pre-weighted to improve the signal to noise ratio. In a frame blocking stage, the segmented PCG signals are blocked into frames using a window length of 30 ms with a 20 ms window overlapping. For a sampling frequency of 44.1 kHz, a hamming window of length 1323 samples was chosen to avoid the parasitic spectral leakage. Fast Fourier transform (FFT) is applied to segmented PCG signals to transform each frame to its frequency domain version. The frequency-domain segmented PCG array is filtered by a group of band-pass Mel triangular filters and transformed into the Mel inverse spectrum domain. The logarithm of Mel spectrum coefficients from each Mel filter is used to compress the higher band of the PCG signal. In the final stage, the logarithmic Mel spectrum coefficients are transformed using the discrete cosine transform (DCT) illustrated in Equation (12).

(12) $$c\left[n\right]=\sum_{m=0}^{N-1}S\left[m\right]\;\cos\left(\frac{\pi n}{M}\left(m-\frac{1}{2}\right)\right),\;n=0,1,2...,M$$

where *M* is the total number of filter banks. For this study, 13 MFCCs were extracted from denoised heart sound.

### 2.5. Feature Fusion

MFCC and 1D-LTP features extracted in previous steps were fused to construct a joint feature vector having dimensions of 1×33. A combination of temporal and frequency features helps in extracting more discriminant information embedded in the PCG signal about heart disorders. Feature fusion is realized through a simple serial concatenation of MFCC and 1D-LTP features.

### 2.6. Classification—Support Vector Machines

The final feature vector from the PCG signal consists of a total of 33 features (20 LTPs + 13 MFCC). Features are extracted from each class (normal, ASD, VSD). The SVM classifier is a widely applied method of classification for biomedical signals [62,63,64,65] due to its excellent generalization capability. It obtains the optimal separating hyperplane for class separation by converting input features to higher dimensions through some nonlinear mapping [66]. The distance between patterns and the hyperplane is maximized using a maximum margin principle to get the best separation. Kernel functions, such as quadratic, cubic, and Gaussian ones, are used for mapping the data into higher dimensional space. Table 3 presents the parameters of classifiers used during training/testing. In this study, SVM was used in two different settings: (1) Binary SVM where input PCG features were labeled as “normal” and “abnormal.” (2) Multiclass experiments where input PCG features were labeled as "normal" or according to the disease type; i.e., ASD or VSD.

## 3. Results

**Figure 9:**
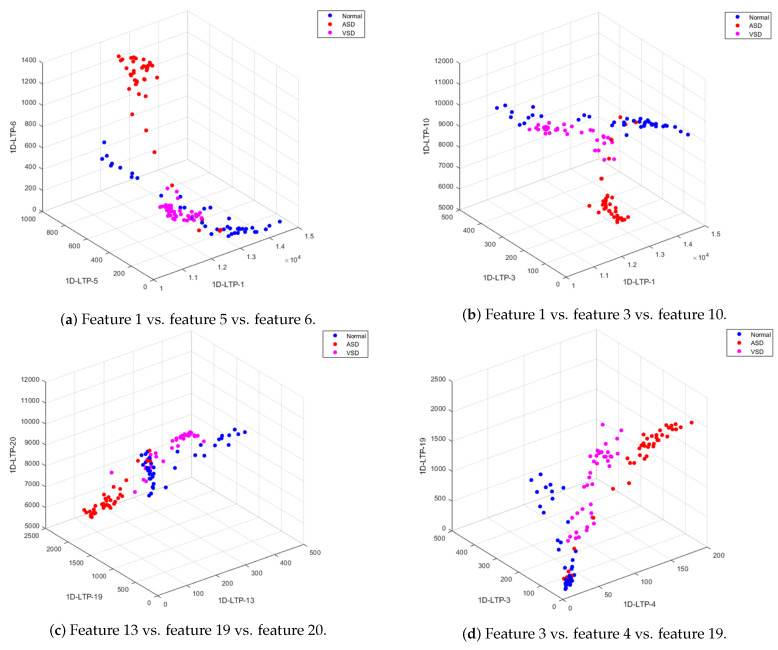
Scatter plots of 1D-LTP features.

In this study, an automated heart disease classification system using the PCG signal is proposed. Raw PCG signal was first preprocessed through EMD, followed by feature extraction through the fusion of MFCC and 1D-LTP features. 1D-LTPs extract the most discriminative information embedded in the PCG signal. Distribution of 1D-LTP features of different classes (normal/ASD/VSD) can be visualized from scatter plots shown (Figure 9). It can be observed that the intra-class difference between features is minimal, while the inter-class difference is maximal. This shows that the extracted features contain generous decisive information about different classes of PCG signals.

The performance of the proposed method was evaluated using standard statistical indices of accuracy, sensitivity (sen), and specificity (spec), which were calculated from the following four parameters

- True positive (TP): abnormal PCG signal correctly detected as abnormal.
- False negative (FN): PCG signal of an abnormal subject detected as normal.
- True negative (TN): normal PCG signal correctly detected as normal.
- False positive (FP): PCG signal of a normal subject detected as abnormal.

(13) $$Accuracy=\frac{TP+TN}{\left(TP+TN+FP+FN\right)}\times 100$$

(14) $$Sen=\frac{TP}{\left(TP+FN\right)}\times 100$$

(15) $$Spec=\frac{TN}{\left(TN+FP\right)}\times 100$$

In this study, the experiments were performed for two different problems.

1. Detection experiment (normal vs. abnormal): All feature vectors belonging to abnormal subjects (ASD, VSD) were labeled as abnormal.
2. Multiclass evaluation (normal vs. ASD vs. VSD): Feature data were labeled according to the disease type in the experiment.

Training and testing of classifiers were pursued through a 10-fold cross-validation method with each subset of features; i.e., MFFC, 1D-LTPs, and fusion of MFCC+1D-LTP. All simulations were performed in MATLAB 2018a on the core i5 computer. All results presented in this paper were averaged over 100 experiments.

### 3.1. Detection Experiment

The experiments for the detection of normal and abnormal subjects were performed on the self-collected dataset using a low-cost data acquisition setup. In detection experiments, the dataset was split into two classes; namely, normal and abnormal. All features vectors belonging to ASD and VSD patients were labeled as abnormal. An SVM classifier with different kernel functions, such as SVM-linear (SVM-L), SVM-quadratic (SVM-Q), SVM-cubic (SVM-C), and SVM-Gaussian (SVM-G), was employed to perform classification. The results of these experiments in terms of accuracy, sensitivity, specificity, positive predictive value (PPV), negative predictive value (NPV), and error rate are illustrated in Table 4. Results of applying individual feature sets (MFCC and 1D-LTP) on PCG signal data are also presented (Table 4). The highest results using only MFCC features were achieved through SVM-C (94.05%); 1D-LTP-only feature extraction achieved the highest accuracy of 94.05% with the SVM-Q classifier. The best results of 95.8% accuracy with SVM-C classifiers were acquired upon feature fusion of MFCCs and 1D-LTPs. Table 5 illustrates the confusion matrix showing individual class accuracy with SVM-C and a combination of MFCC and 1D-LTP features. It was evident from experimentation that the fusion of MFCC and 1D-LTP features provide a significant improvement in classification performance.

### 3.2. Multiclass Evaluation (Normal vs. ASD vs. VSD)

Multiclass experiments were performed to precisely identify the type of heart disorder. Features were labeled according to the disorder type; i.e., ASD, VSD, or normal. A multiclass SVM with different kernels was trained and tested using 10-fold cross-validation. The results of applying different multiclass SVM classifiers on individual feature sets (MFCC, 1D-LTP) and the fusions of both are illustrated in Table 6. The obtained results revealed that the SVM-C classifier achieved a peak accuracy of 88.69% with only MFCC features, while the same classifier provided 94.64% accuracy with 1D-LTP features. Performance results were further improved by the fusion of MFCC and 1D-LTP features with the SVM-C classifier; i.e., 95.24% accuracy. In Table 7, class-wise information of accuracy for ASD, VSD, and normal classes in the form of a confusion matrix with the SVM-C classifier are shown. The proposed feature fusion methodology effectively extracted the characteristic information from multiclass PCG signals.

### 3.3. Statistical Significance

The primary objective behind performing this statistical analysis was to achieve a certain level of confidence in the proposed scheme. Analysis of variance (ANOVA) [67] was utilized to testify whether the results were statistically significant or not—simply by comparing the means of multiple distributions.

In this work, a proposed scenario (MFCC + 1D-LTP) was considered for two different classifiers (SVM-C, SVM-Q)—selected based on the improved performance compared to the rest. In using ANOVA, a series of tests were performed for the assumptions of normality and homogeneity of variance. A Shapiro–Wilk test [68] was performed for the former, and the Bartletts test [68] for the latter one—with the significance level α selected to be 0.01. The means of our approach were $\bar{x_{1}}$,$\bar{x_{2}}$, calculated from the overall accuracy of both classifiers. The null hypothesis $H_{0}$, given that $\bar{x_{1}}=\bar{x_{2}}$, while the alternative hypothesis $H_{a}$ given that $\bar{x_{1}}\neq \bar{x_{2}}$. The *p*-value was computed and the null hypothesis was tested, $H_{0}$; if it was rejected, p<α, then the Bonferroni posthoc test was applied.

**Figure 10:**
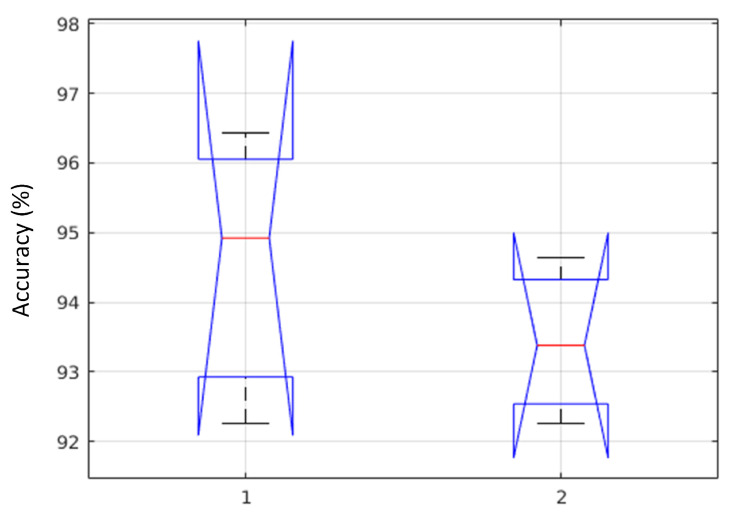
Box-plot of accuracy values for selected classifiers (1:SVM-C, 2:SVM-Q).

For the proposed method (MFCC + 1D-LTP), and with selected classifiers (SVM-C and SVM-Q), the Shapiro–Wilk test generated *p*-value, $p_{c}=0.6987$, and $p_{q}=0.9352$. By following the Bartletts test, the associated chi-squared probabilities were: $p_{c}=0.712$ and $p_{q}=0.312$. The *p*-values of two different classifiers are significantly greater than α. Therefore, from the test results (normality and equality of variances), we failed to repudiate the null hypothesis $H_{0}$, and we are confident in claiming that the test data were normally distributed, and the variances were also homogeneous. The ANOVA test, including five different parameters (degrees of freedom (dfs), a sum of squared deviation (SS), mean squared error (MSE), F-statistics, and *p*-value) is shown in Table 8. The performance ranges of two selected classifiers based on the proposed method are shown in Figure 10.

**Figure 11:**
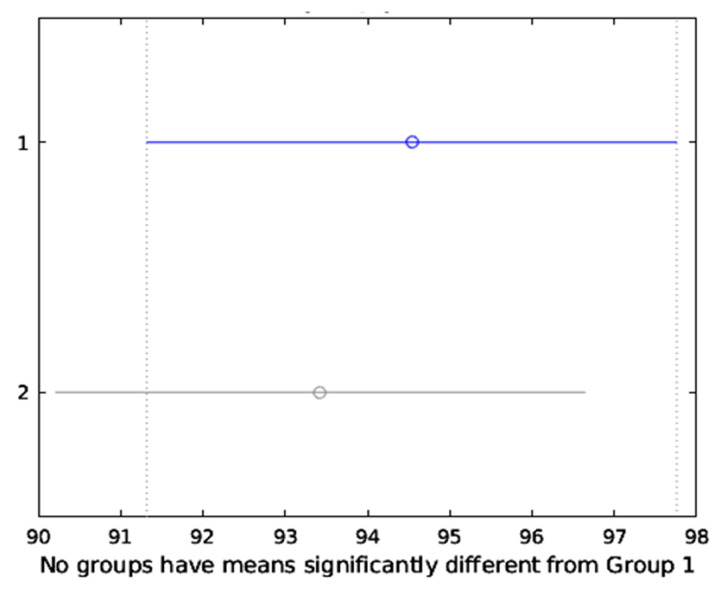
The means of both classifiers belong to a single group and are not significantly different.

The results were validated based on the Bonferroni post hoc test, Figure 11, which is the most common approach to be applied whenever there exists a chance of a significant difference between the means of multiple distributions. It was certified that the proposed method performed much better than conventional methods.

## 4. Discussion

The proposed method of feature fusion with EMD-based signal denoising effectively extracted embedded information from PCG signals using the self-collected dataset of ASD and VSD cardiac disorders. The MFCC extracted frequency-domain features, while 1D-LTP features extracted temporal and texture information from the signal. Feature fusion of these two different types provided a powerful signal representation for different classes (normal, ASD, VSD) with a high degree of accuracy. Moreover, the proposed method classified normal and abnormal PCG data through SVM-C classifier with 95.83% accuracy, while 95.34% average accuracy was achieved on multiclass PCG data with the same classifier.

The numbers of classes, feature extraction techniques and classification methods of the proposed method were compared with the previously developed platforms (Table 1), which showed that several existing works [9,10,11,13,15,17] utilized the Physionet Challenge 2016 dataset [69] comprised of only two classes (healthy and unhealthy) while others used self-collected PCG signal data. MFCCs were widely employed by several studies [9,11,17,35], and acted as baseline features of choice. The SVM classifier is also widely adopted by existing works [10,11,12,13].

DWT and statistical features were used with a multilayer perceptron to achieve 96.6% accuracy on normal and ASD classes of PCG data [36]. In another work [38], a comparison of feature reduction methods was demonstrated. Experimental results are shown between normal and three different classes of heart diseases; i.e., ASD, VSD, and aortic stenosis. Feature reduction methods (DDE, FSDA, CBEF, EFUAP) were applied with K-nearest neighbor (KNN) classifier and 84.3% accuracy was achieved.

**Figure 12:**
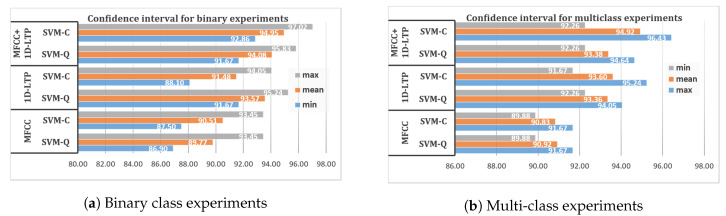
Confidence interval simulation results.

In contrast to the existing work, our research targeted the classification of multiple heart disorders (ASD, VSD) with the feature fusion approach of MFCC and new temporal feature descriptor 1D-LTP. The proposed method outperforms the existing approaches, as is evident from the presented results. To confirm the validity and robustness of our proposed method, confidence intervals against binary and multiclass experiments are also provided for the two best classifiers; i.e., SVM-C and SVM-Q. Figure 12a illustrates the confidence interval showing maximum, minimum, and average classification results of individual MFCC and 1D-LTP features and the feature fusion approach for binary experiments. Figure 12b presents a confidence interval of minimum, maximum, and average classification accuracy for multiclass experiments. From this comprehensive statistical analysis, it is quite straightforward to choose SVM-C as a standard classifier for this application.

## 5. Conclusions

Preprocessing and classification of heart sounds is a challenging problem due to the addition of environmental noise. The addition of noise may hide the actual class information in the PCG signal. In this study, an effective classification framework was developed for the diagnosis of ASD, VSD, and normal subjects through PCG signal analysis. A feature fusion approach using novel 1D-LTP features along with strong MFCC features has shown to be an effective strategy exhibiting good discriminative properties of representing PCG signals. The proposed method was validated through different SVM kernels, and the best performance was achieved with SVM-C. The main findings of this research are the following:

- The proposed framework is non-invasive and reliable.
- The proposed scheme is independent of the morphological characteristics of the acquired PCG signal.
- This research introduces a new feature descriptor, i.e., 1D-LTP, that significantly improves the classification performance upon fusion with classical MFCCs.
- The proposed method is fully automated and works with all kinds of noisy PCG signals due to the adoption of a data-driven preprocessing approach; i.e., EMD.

This research has the following shortcomings:

- The dataset used is small in size.
- The selection of proper IMFs in EMD is not automated.

The proposed method for cardiac disorders can be enhanced by adding more data samples of PCG. In the future, we aim to apply feature reduction and fusion algorithms to further reduce the feature vector dimensions and increase system accuracy.

## 6. Compliance with Ethical Standards

### 6.1. Ethical Approval

All procedures performed in studies involving human participants were in accordance with the ethical standards of the institutional and/or national research committee and with the 1964 Helsinki declaration and its later amendments or comparable ethical standards.

### 6.2. Informed Consent

Informed consent was obtained from all individual participants included in the study.

## Figures and Tables

**Table 1:** Comparison with existing literature.

Ref	Year	Dataset	Classes	Features	Classifier	Results
[9]	2016	Physionet Challenge 2016 [39]	Normal(2488), Abnormal(665)	Time-frequency, Wavelet and statistical	LogitBoost, Random Forest	Acc: 84.48%
[11]	2016	Physionet Challenge 2016 [39]	Normal(2575), Abnormal(665)	Dynamic time warping	SVM	Acc: 82.4%
[15]	2016	Physionet Challenge 2016	Normal(2575), Abnormal(665)	124 Time-frequency features	Adaboost, CNN	Acc: 89%
[12]	2016	Self-collected	Normal(132), Abnormal seven classes(131)	Arash-Band	SVM	Acc: 87.45%
[36]	2017	Self-collected	Small VSD(60), Large VSD(60)	Statistical, DWT features	Multilayer Perceptron (MLP)	Acc: 96.6%
[35]	2017	Self-collected	Normal, VSD	(STFT), MFCC	KNN	Acc: 93.2%
[13]	2018	PhysioNet Computing in Cardiology Challenge	Normal(2575), Abnormal(665)	GFCC	Weighted SVM	Sen: 90.3% Spec: 89%
[17]	2018	UoC-murmur database, PhysioNet-2016	Normal(336), CHD(130), Normal/Abnormal(2435)	Mel-Spectrogram, MFFC and sub-band envelopes	CNN	Acc: 81.5% Sen: 84.5%
[10]	2018	PhysioNet Computing in Cardiology Challenge-2016	Normal(50), Abnormal(50)	Cepstrum Analysis	SVM	Acc: 95%
[38]	2018	Self-collected	Normal(40), Abnormal(58)	CBFE, FEUAP, FSDA, DDE	KNN	Acc: 84.39%
[32]	2019	Self-collected	Normal(175), Abnormal(108)	MFCC, normalized average Shannon energy	SVM	Acc: 92.6%
**This work**	**2020**	**Self-collected**	**Normal(140), Abnormal(140)**	**MFCC + 1D-LTPs**	**SVM**	**Acc: 95.63%**
**This work**	**2020**	**Self-collected**	**Normal(140), ASD(85), VSD(55)**	**MFCC + 1D-LTPs**	**SVM**	**Acc: 95.24%**

**Table 2:** Description of PCG dataset.

Status	No. of Signals	No. of Subjects	Male	Female
Normal	140	28	17	11
ASD	85	17	12	5
VSD	55	11	7	4

**Table 3:** Parameters of selected classifiers.

Classifier	Kernel Function	Kernel Scale	Box Constraint Level	Multiclass Method	Standardize Data
**SVM-L**	Linear	Automatic	1	One-vs-one	True
**SVM-Q**	Quadratic	Automatic	1	One-vs-one	True
**SVM-C**	Cubic	Automatic	1	One-vs-one	True
**SVM-G**	Gaussian	44	1	One-vs-one	True

**Table 4:** Performance comparison of SVM on different feature sets for binary experiments. Bold font indicates the best result obtained against each feature set.

Feature Set	Classifier	Accuracy (%)	Sensitivity (%)	Specificity (%)	PPV (%)	NPV (%)	Error (%)
**MFCC**	SVM-L	89.88	76.19	94.44	82.05	92.25	10.12
	SVM-Q	89.29	80.95	92.06	77.27	93.55	10.71
	**SVM-C**	**92.26**	**88.1**	**93.65**	**82.22**	**95.93**	**7.74**
	SVM-G	75.6	7.14	98.41	60	76.07	24.4
**1D-LTP**	SVM-L	94.05	88.1	96.03	88.1	96.03	5.95
	**SVM-Q**	**94.05**	**83.33**	**97.62**	**92.11**	**94.62**	**5.95**
	SVM-C	91.07	76.19	96.03	86.49	92.37	8.93
	SVM-G	86.31	47.62	99.21	95.24	85.03	13.69
**MFCC+1D-LTP**	SVM-L	94.05	90.48	95.24	86.36	96.77	5.95
	SVM-Q	94.05	88.1	96.03	88.1	96.03	5.95
	**SVM-C**	**95.83**	**92.86**	**96.83**	**90.7**	**97.6**	**4.17**
	SVM-G	93.45	88.1	95.24	86.05	96	6.55

**Table 5:** Confusion matrix for detection (normal vs. abnormal) experiments.

	Predicted Class	
Actual Class	Normal	Abnormal
**Normal**	90%	10%
**Abnormal**	2%	98%

**Table 6:** Performance comparison of SVM using different feature sets for multiclass experiments. Bold font indicates the best result obtained against each feature set.

Feature Set	Classifier	Accuracy(%)	Sensitivity(%)	Specificity(%)	PPV(%)	NPV(%)	Error(%)
**MFCC**	SVM-L	83.93	92.86	85.71	68.42	97.3	16.07
	SVM-Q	86.9	90.48	90.48	76	96.61	13.1
	**SVM-C**	**88.69**	**90.48**	**94.44**	**84.44**	**96.75**	**11.31**
	SVM-G	83.33	97.62	81.75	64.06	99.04	16.67
**1D-LTP**	SVM-L	94.64	97.62	93.65	83.67	99.16	5.36
	SVM-Q	94.05	90.48	95.24	86.36	96.77	5.95
	**SVM-C**	**94.64**	**90.48**	**96.03**	**88.37**	**96.8**	**5.36**
	SVM-G	93.45	92.86	93.65	82.98	97.52	6.55
**MFCC+1D-LTP**	SVM-L	93.45	97.62	92.06	80.39	99.15	6.55
	SVM-Q	94.43	95.05	94.41	85.06	98.28	5.57
	**SVM-C**	**95.24**	**95.24**	**95.24**	**86.96**	**98.36**	**4.76**
	SVM-G	93.45	100	91.27	79.25	100	6.55

**Table 7:** Confusion matrix for multiclass experiments.

	Predicted Class		
Actual Class	Normal	ASD	VSD
**Normal**	90%	10%	0%
**ASD**	6%	94%	0%
**VSD**	0%	0%	100%

**Table 8:** ANOVA test on two selected classifiers based on the proposed method.

Variance Source	SS	df	MSE	F-Statistics	*p*-Value
**Between**	1.8482	1	1.84815	0.63	0.4721
**Within**	11.7503	4	2.93758	-	-
**Total**	13.5985	5	-	-	-
